# Estimated Daily Phthalate Exposures in a Population of Mothers of Male Infants Exhibiting Reduced Anogenital Distance

**DOI:** 10.1289/ehp.8663

**Published:** 2006-02-02

**Authors:** Kevin Marsee, Tracey J. Woodruff, Daniel A. Axelrad, Antonia M. Calafat, Shanna H. Swan

**Affiliations:** 1 Joint Medical Program, School of Public Health, University of California, Berkeley, California, USA; 2 Office of Policy, Economics, and Innovation, U.S. Environmental Protection Agency, San Francisco, California, USA; 3 Office of Policy, Economics, and Innovation, U.S. Environmental Protection Agency, Washington, DC, USA; 4 Division of Laboratory Sciences, National Center for Environmental Health, Centers for Disease Control and Prevention, Atlanta, Georgia, USA; 5 Department of Obstetrics and Gynecology, University of Rochester, Rochester, New York, USA

**Keywords:** anogenital distance, butyl-benzyl phthalate, di(2-ethylhexyl) phthalate, dibutyl phthalate, diethyl phthalate, diisobutyl phthalate, exposure estimates, reference dose

## Abstract

Phthalate diesters have been shown to be developmental and reproductive toxicants in animal studies. A recent epidemiologic study showed certain phthalates to be significantly associated with reduced anogenital distance in human male infants, the first evidence of subtle developmental effects in human male infants exposed prenatally to phthalates. We used two previously published methods to estimate the daily phthalate exposures for the four phthalates whose urinary metabolites were statistically significantly associated with developmental effects in the 214 mother–infant pairs [di-*n*-butyl phthalate (DnBP), diethyl phthalate (DEP), butylbenzyl phthalate (BBzP), diisobutyl phthalate (DiBP)] and for another important phthalate [di-2-ethylhexyl phthalate (DEHP)]. We estimated the median and 95th percentile of daily exposures to DBP to be 0.99 and 2.68 μg/kg/day, respectively; for DEP, 6.64 and 112.3 μg/kg/day; for BBzP, 0.50 and 2.47 μg/kg/day; and for DEHP, 1.32 and 9.32 μg/kg/day. The U.S. Environmental Protection Agency (EPA) reference doses for these chemicals are 100 (DBP), 800 (DEP), 200 (BBzP), and 20 (DEHP) μg/kg/day. The median and 95th percentile exposure estimates for the phthalates associated with reduced anogenital distance in the study population are substantially lower than current U.S. EPA reference doses for these chemicals and could be informative to any updates of the hazard assessments and risk assessments for these chemicals.

Phthalates are used in a variety of industries and are present in many consumer products, such as soaps, perfumes, cosmetics, shampoos, building products, shower curtains, aerosols, plastic toys, and plastic packaging [Agency for Toxic Substances and Disease Registry (ATSDR) 1995, 2001, 2003]. Di(2-ethylhexyl) phthalate (DEHP) is the primary plasticizer in polyvinyl chloride, and diethyl phthalate (DEP) and dibutyl phthalates (DBPs) are commonly used in consumer and personal care products such as lotions, fragrances, cosmetics, deodorants, and pharmaceutical coatings (ATSDR 1995, 2001, 2003). The reproductive and developmental toxicities of some phthalates have been demonstrated extensively in animal studies. Prenatal exposure to DEHP, DBP, butyl-benzyl phthalate (BBzP), or, more weakly, diisononyl phthalate reduces testosterone production in fetal testes (Lehmann et al. 2004; Mylchreest and Foster 2000; Mylchreest et al. 2002; Parks et al. 2000), which can result in incomplete development of the male reproductive tract and malformations of the external genitalia (Ema and Miyawaki 2001; Ema et al. 2003; Foster et al. 2000; Gray et al. 2000; Mylchreest et al. 1998).

In a study published in a previous issue of this journal by some authors participating in the current study, Swan et al. (2005) provided the first demonstration of subtle developmental effects, similar to those seen in animal studies, in human male infants exposed prenatally to phthalates. The study population for Swan et al. (2005), described below, included 134 women whose male offspring had a physical examination by 17 December 2004, of whom 85 had also given a urine sample during pregnancy. These prenatal maternal urine samples were analyzed for nine phthalate metabolites commonly used as biomarkers of exposure to phthalates, using an analytical method described before (Silva et al. 2004b). One hundred thirty-four male infants, including 49 for whom no maternal prenatal urine sample had been collected, were physically examined to determine anogenital distance (AGD)—a marker for prenatal antiandrogen exposure—and other reproductive organ measurements. Of nine urinary phthalate metabolites, Swan et al. (2005) found that prenatal maternal urinary levels of monoethyl phthalate (MEP; a metabolite of DEP), monobenzyl phthalate (MBzP; a metabolite of BBzP), mono-*n*-butyl phthalate [MBP; a metabolite of di-*n*-butyl phthalate (DnBP)], and monoisobutyl phthalate [MiBP; a metabolite of diisobutyl phthalate (DiBP)] were significantly associated with reduced AGD and anogenital index (AGI = AGD/body weight) in male infants.

Although none of the 134 boys examined showed frank malformations or disease, and 86.6% of these boys had both testicles classified as normal, AGI was significantly correlated with degree of testicular descent as well as penile volume and scrotal size (Swan et al. 2005). The median concentrations of phthalate metabolites (Table 1) in the Swan et al. study associated with short AGI and incomplete testicular descent were similar to the median concentrations found in the female population of the United States, based on the 2001–2002 National Health and Nutrition Examination Survey (NHANES) (National Center for Environmental Health 2005).

The current U.S. Environmental Protection Agency (EPA) reference doses (RfDs) for DEP, DBP, and BBzP were formulated in the early 1990s using older animal studies (DBP was completed in 1990; DEP and BBzP in 1993) (U.S. EPA 2005a, 2005c, 2005d). The RfD, as defined by the U.S. EPA, is intended to be a dose for which daily oral exposure to the human population (including sensitive subgroups) is likely to be without an appreciable risk of deleterious effects during a lifetime. Because the data presented by Swan et al. (2005) suggested subtle human developmental effects at levels of exposure similar to those observed in the general population, that study may provide important information when considering any future updates to RfDs for phthalates. For that study to be useful for this purpose, it is necessary to estimate the average daily exposures of phthalates for the study individuals.

In this study, we applied a simple pharmacokinetic model, initially proposed by Kohn et al. (2000) and later used by Koo et al. (2002), to estimate the individual daily exposure of phthalate diesters in the pregnant women in the Swan et al. (2005) study population. We also used a second model, initially proposed by David (2000), to provide comparisons for our exposure estimates generated by the first model.

## Materials and Methods

### Study population

Women included in this study were originally recruited into the Study for Future Families (SFFI), a multicenter pregnancy cohort study, at prenatal clinics in Los Angeles, California (Harbor-UCLA and Cedars-Sinai); Minneapolis, Minnesota (University of Minnesota Health Center); and Columbia, Missouri (University Physicians), from September 1999 through August 2002. Details of study participation are given by Swan et al. (2005). All participants completed a questionnaire, and after urine collection was added midway through the study, most gave a urine sample. Eighty-five percent of SFFI participants agreed to be recontacted, and these mothers were invited to take part in the SFF follow-up study (SFFII) (Swan et al. 2005). Human subject committees at all participating institutions approved the SFFI and SFFII, and all participants signed an informed consent for each study.

In the Swan et al. (2005) study, the authors reported on results in boys for whom a first pre-natal visit had been completed by 17 December 2004. These included 172 boys, 134 of whom had complete data for AGD, age, and weight. Urinary phthalate metabolite concentrations in 214 mother–infant pairs were also obtained (girls and boys), of whom 85 were boys with measurements of AGD and complete data on age and weight and whose mother had given a prenatal urine sample. We used the urinary phthalate monoester concentrations from the study population of 214 mother–infant pairs to calculate daily exposure estimates. The monoester concentrations for the complete study population (*n* = 214) are shown in Table 1. This study population has urinary monoester concentrations very similar to those found in the subset of this population (*n* = 85) used by Swan et al. (2005). Distributions of phthalate metabolites among the groups of 85 and 214 women are similar. The median monoester concentrations in the group of 85 were 128.4 ng/mL (MEP), 13.5 ng/mL (MBP), 8.3 ng/mL (MBzP), 2.5 ng/mL (MiBP), 3.3 ng/mL (MEHP), 11.4 ng/mL (MEHHP), and 11.1 ng/mL (MEOHP). We evaluated the larger sample because it provides more information on the distribution of phthalate exposures.

We calculated daily exposure for the phthalate metabolites that were statistically significantly associated with reduced AGI in the Swan et al. (2005) study, and the metabolites of DEHP. Although metabolites of DEHP were not significantly associated with AGI in the Swan et al. study, the associations for two oxidative metabolites of DEHP [mono(2-ethyl-5-oxohexyl) phthalate (MEOHP) and mono(2-ethyl-5-hydroxyhexyl) phthalate (MEHHP)] were of magnitudes comparable with those for metabolites of DBP and BBzP. Moreover, there is an extensive animal literature showing DEHP-mediated androgen-related effects.

### Daily exposure estimates

Kohn et al. (2000) calculated the daily exposure for each individual in the population by using a linear two-compartment model. The normalized integrated rate equations for fractional excretion are as follows:


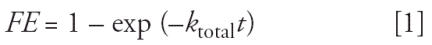



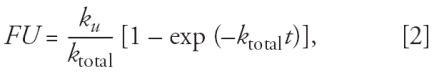


where *FE* is the total fraction and *FU* is the urinary fraction of the dose eliminated in time *t*, and *k*_total_ and *k_u_* are the apparent first-order rate constants for total elimination and urinary elimination of monoester, respectively. We calculated the two rate constants, *k*_total_ and *k_u_*, by using previously published values for the excreted fractions of each parent diester (Kohn et al. 2000; Koo et al. 2002). Values of *FE* and *FU* from Kohn et al. (2000), originally calculated from animal and human studies, were used for all metabolites reported by Swan et al. (2005), except for MiBP, which was not considered by Kohn et al. We assumed that the *FE* and *FU* for MiBP and its parent diester were equal to those calculated for MBP and DnBP. The excretion rate equations are used to estimate *k*_total_ and *k_u_* for input into the equation from Kohn et al. (2000) that estimates phthalate exposure.

Kohn et al. (2000) provide the following equation for the exposure rate for an individual, assuming steady-state exposure and metabolic clearance of the diester:


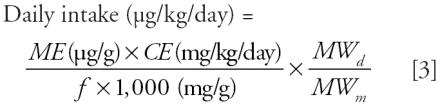


where *ME* is the urinary concentration of monoester per gram of creatinine, *CE* is the creatinine excretion rate normalized by body weight, *f* is the ratio of urinary excretion to total elimination (*k_u_*/*k*_total_), and *MW_d_* and *MW_m_* are the molecular weights of the diesters and monoesters, respectively. We used a value of 18 mg/kg/day for *CE* (Kohn et al. 2000) and creatinine-adjusted concentrations (*ME*) for each subject in the study. The unadjusted and creatinine adjusted phthalate urinary concentrations from the 214 samples from the Swan et al. (2005) study are shown in Table 1.

For comparison, we also estimated the daily exposure using a second formula published by David (2000), and later used by Koch et al. (2003):


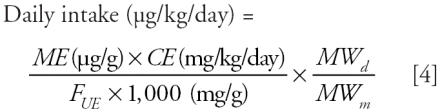


This formula is an alternate version of the method of Kohn et al. (2000) and results in similar exposure values (Koch et al. 2003). The variables used are the same as those used in the Kohn et al. formula, except *F_UE_*, the molar fraction of the urinary excreted monoester related to the parent diester, is used in place of *f.* The fractional urinary excretion values for DBP (0.69) and BBzP (0.73) were taken from published human data (Anderson et al. 2001). For DEP, we presumed the excretion factor to be the same as that for DBP, as done by Koch et al. (2003) and Kohn et al. (2000). The fractional excretion data for the three DEHP metabolites measured in the Swan et al. (2005) study were taken from recently published human data (Koch et al. 2005). The values for mono(2-ethylhexyl) phthalate (MEHP), MEHHP, and MEOHP are 0.059, 0.233, and 0.150, respectively.

We calculated DEHP exposures based on each of the three metabolites independently, and also based on the averages of the exposures calculated using the secondary metabolites (MEHHP and MEOHP). DEHP is initially metabolized to MEHP, which is then further metabolized to various other products, including MEHHP and MEOHP. All three metabolites are thought to be toxic (Koch et al. 2005), and estimating DEHP exposure based on the three different metabolites allows for comparison of the various estimates. We treated the concentrations of MBP and MiBP as one combined measure of exposure to DBP. This makes it consistent with previous literature on DBP, which did not distinguish between iso- and *n*-butyl isomers. We considered MBzP to be the main metabolite of BBzP. MBP is a minor metabolite of BBzP, but only 6% of the ingested BBzP diester is excreted as MBP (Anderson et al. 2001).

## Results

The results for the exposures of DEP, DBP, and BBzP of the women in the Swan et al. (2005) study, as calculated using the Kohn et al. (2000) method, are presented in Table 2. The relevant monoesters are presented with their parent diesters. Using the Kohn et al. formula, we estimated the median and 95th percentile of daily exposures for DBP to be 0.99 and 2.68 μg/kg/day, respectively; DEP, 6.64 and 112.3 μg/kg/day; BBzP, 0.50 and 2.47 μg/kg/day; and DEHP, 1.32 and 9.32 μg/kg/day.

The estimated exposures calculated using the David (2000) formula are compared with the Kohn et al. (2000) method estimates in Table 3. The David method produces exposure estimates that are typically about 20% lower. The exception is DEHP, which is about 30–80% higher based on the David method, depending on which metabolites are used for the calculation.

## Discussion

We have estimated exposures to a variety of phthalate diesters in a population of mother–infant pairs in which subtle developmental effects were associated with prenatal urinary phthalate metabolite concentrations. The models we have used to estimate exposures make no assumptions regarding the route of exposure. There are multiple possible routes of exposure to phthalates, including dermal (Duty et al. 2005), ingestion (Clark et al. 2003), and inhalation (Adibi et al. 2003). Furthermore, phthalate diesters and their metabolites are cleared from the body within a few days, with the bulk of the dose cleared within the first 24 hr (Anderson et al. 2001; Koch et al. 2005). There were relatively few nondetects in the population, indicating that exposures of the levels observed in this study reflect relatively continuous daily exposures.

The median estimated exposures for DBP and BBzP in the Swan et al. (2005) study population (*n* = 214) are on the order of 1 μg/kg/day, and for DEP are on the order of 6 μg/kg/day. Current U.S. EPA RfDs are 100 μg/kg/day (DBP), 200 μg/kg/day (BBzP), and 800 μg/kg/day (DEP), which are all more than 100 times greater than the median exposures in the Swan et al. (2005) population.

There are potential sources of uncertainty in the Kohn et al. (2000) and David (2000) formulas. Creatinine excretion rates are known with 10% accuracy (Kohn et al. 2000). Furthermore, Kohn et al. discuss the potential uncertainty within the total and urinary excretion values (*FE* and *FU*). Because Kohn et al. used animal excretion data for some of the metabolites, they estimated that their *FE* values were accurate to approximately 50%, whereas the *FU* values could vary by 15-fold among species with humans in the middle. However, we used fractional urinary excretion values obtained from human studies in our calculations using the David formula. There has been much scientific debate regarding the appropriate use of *F_UE_* values when using the David formula to calculate DEHP exposure values. David (2004) has argued in favor of using an *F_UE_* for MEHP of 13%, calculated from human excretion data provided by Anderson et al. (2001). In a reply to David (2004), Koch et al. (2004) support their use of an *F_UE_* for MEHP of 2.4% and also provide a mathematical argument against the feasibility of 13% as the *F_UE_* for MEHP. The choice of *F_UE_* values is important because it affects the results of the exposure calculations. We use *F_UE_* values from the most recent human excretion data on DEHP (Koch et al. 2005). Our MEHP *F_UE_* of 5.9% falls in between the values previously proposed by David and Koch et al. Our exposure calculations using this value are in close agreement with our calculations using the oxidative metabolites of DEHP (MEHHP and MEOHP), and with our calculations using the Kohn et al. method, which does not use *F_UE_* values. Our exposure estimates from the Kohn et al. and David formulas are similar, suggesting reasonable agreement between the models and parameters used.

The exposures within this study population of pregnant women are similar to or somewhat lower than those documented in other populations of women of reproductive age. Median female (all ages above 6 years) MBP concentrations in NHANES 2001–2002 (21.5 μg/g creatinine) compare closely with those in the Swan et al. (2005) study population (20.6 μg/g creatinine), and median 2001–2002 NHANES MBzP concentrations (15.1 μg/g creatinine) were similar to those in the Swan et al. study population (11.7 μg/g creatinine) (National Center for Environmental Health 2005). In the population of 97 women 20–40 years of age that was evaluated in the Kohn et al. (2000) study, the median concentrations of MBP, MBzP, MEP, and MEHP were greater than those in the Swan et al. study population. A population of 25 pregnant women in New York City exhibited median MBP, MEP, and MBzP urinary concentrations within the same order of magnitude but higher than those observed in the Swan et al. study population (Adibi et al. 2003).

Except for the Adibi et al. (2003) study, the studies mentioned above deal primarily with women who are not pregnant, whereas the women in the Swan et al. (2005) study population were pregnant. Differences in fluid level and metabolism between pregnant and nonpregnant states may account for some of these differences. Alternatively, the differences among the study populations may represent temporal differences in exposures to phthalate-containing materials. The Kohn et al. (2000) study evaluated samples collected from 1988–1994 (NHANES III), whereas the Adibi et al. (2003) study evaluated samples collected in 2000. Samples from the Swan et al. (2005) study population were collected from 2000 through 2003.

As discussed in the Swan et al. (2005) study, the observed relationships between pre-natal phthalates and AGD in male infants are similar to those observed in animal studies, in which those changes are seen only at higher doses (Swan et al. 2005). For DBP, Mylchreest et al. (2000) found androgen-dependent effects from exposure in rats, such as decreased AGD, retained areolas or nipples, and reproductive tract malformations. The most sensitive end point observed was a dose-dependent increase in the incidence of thoracic areola and nipple development. When compared with the control animals, the lowest statistically significant dose group was at 100 mg/kg/day (100,000 μg/kg/day). This is well above the values obtained from the Swan et al. study. Some of the difference could be attributed to the difference in study design, in which Mylchreest et al. compared each dose group only with the controls and did not present an overall test for trend among the doses, in contrast to the Swan et al. study, which looked at a continuous dose response function. It may also suggest that humans could be more sensitive than animals to exposures to phthalates. A separate study (Lehmann et al. 2004) demonstrated statistically significantly reduced fetal testicular testosterone production with daily DnBP administration as low as 50 mg/kg/day in experimental rats. Alterations to the activity of enzymes involved in the production of testosterone were observed at DnBP levels as low as 0.1 mg/kg/day. Given the small sample size of the study (four to five fetuses per treatment group), it is possible that effects at DnBP doses < 50 mg/kg/day might significantly reduce fetal testosterone production in animal models.

In addition, the observed associations in the Swan et al. (2005) study at the lower concentrations could reflect the “real-world” scenario that occurs in the human population, where exposure to any individual chemical of interest occurs simultaneously with exposures to other environmental factors that could affect the dose at which effects are seen. In the Swan et al. study, multiple phthalates, many of which have androgen-related effects (Gray et al. 2000; Lehmann et al. 2004; Mylchreest et al. 2000; Parks et al. 2000), were detected in the women. In the animal studies, only one chemical is assessed at a time, which cannot account for the effect of multiple exposures that occur in the human population. Recent research with rats dosed with mixtures of chemical antiandrogens, including DBP, DEHP, BBzP, and four different herbicides, indicates that all tested mixtures of these chemicals acted to produce cumulative, apparently dose-additive effects on androgen-dependent tissues (Gray et al. 2006).

Swan et al. (2005) found subtle developmental effects associated with phthalate exposures in a human population. The exposures that are associated with these subtle developmental reproductive effects in male infants are comparable with exposures observed in other female populations in the United States (Adibi et al. 2003; Blount et al. 2000; National Center for Environmental Health 2005; Silva et al. 2004a) and are two orders of magnitude lower than the reference doses assumed to be protective by the U.S. EPA (U.S. EPA 2005a, 2005b, 2005c, 2005d). The values of our exposure estimates are in close agreement when calculated using two different models and different excretion factors. We have provided exposure estimates for the phthalates deemed to have had health effects in this study population. This information is an asset to any future updates of the RfDs for these phthalates.

## Figures and Tables

**Table 1:** Urinary phthalate monoester concentrations (ng/mL urine, μg/g creatinine) from a study population of 214 pregnant women from Swan et al. (2005).^a^

Phthalate	25th percentile	Median	75th percentile	95th percentile	Maximum	NHANES median^b^
MEP						
ng/mL	50	117	466	3,199	30,528	167
μg/g creatinine	71.1	108	506	3,015	33,932	171
MBzP						
ng/mL	3.6	9.3	20.9	57.8	436	15.4
μg/g creatinine	6.5	11.7	21.6	58	364	15.1
MBP						
ng/mL	7.4	16.2	29.6	64.5	337	21.6
μg/g creatinine	13.8	20.6	32.2	57.3	144	21.5
MiBP						
ng/mL	< LOD	2.5	4.7	13.1	39.8	2.50
μg/g creatinine	< LOD	2.9	5.1	10.0	71.1	2.83
MEHP						
ng/mL	1.5	4.25	11.0	38.6	206.8	4.10
μg/g creatinine	2.15	5.53	14.0	39.2	172.8	4.43
MEHHP						
ng/mL	5.6	10.8	21.7	76.4	2,108	18.2
μg/g creatinine	8.4	13.0	26.9	88.9	1,254	17.6
MEOHP						
ng/mL	5.1	9.75	21.0	65.0	1,677	13.0
μg/g creatinine	7.7	12.6	23.1	80.5	998	12.0

LOD, limit of detection.

a Swan et al. (2005) report phthalate concentrations for the 85 infant–mother pairs with sufficient data for the epidemiologic analysis. The present analysis uses the original sample of 214 with urinary metabolite concentrations (see “Materials and Methods”).

b The median concentration in the general female (older than 6 years) population from NHANES 2001–2002 (National Center for Environmental Health 2005).

**Table 2:** Estimated phthalate exposure (μg/kg/day), calculated using the Kohn et al. (2000) method, for 214 pregnant women from Swan et al. (2005).

Monoester	Diester (parent)	25th percentile	Median	75th percentile	95th percentile	Maximum
MEP	DEP	2.65	6.64	18.82	112.3	1,263
MBzP	BBzP	0.28	0.50	0.092	2.47	15.53
MBP	DnBP	0.56	0.84	1.31	2.33	5.86
MiBP	DiBP	NA^a^	0.12	0.21	0.41	2.90
MiBP + MBP	DnBP + DiBP	0.63	0.99	1.53	2.68	5.98
MEHP	DEHP	0.51	1.32	3.32	9.32	41.10

NA, not applicable. The phthalates shown are those that were significantly associated with reduced AGD and AGI (Swan et al. 2005), along with MEHP. Current U.S. EPA RfDs are 100 (DBP), 200 (BBzP), (DEP), and 20 (DEHP) μg/kg/day (U.S. EPA 2005a, 2005b, 2005c, 2005d).

a The daily exposure was not estimated when the urinary concentration of the phthalate metabolite was < limit of detection.

**Table 3:** Estimated daily exposure values of phthalates to the pregnant women of Swan et al. (2005) study population based on the Kohn et al. (2000) and the David^a^ ^2000^ methods.^b^

		Kohn et al. method			David method		
Metabolite	Diester	Median	95th percentile	Range	Median	95th percentile	Range
MEP^c^	DEP	6.64	112.3	0–1,263	5.32	90.0	< LOD to 1,013
MBzP^c^	BBzP	0.50	2.47	0–15.5	0.35	1.74	< LOD to 10.9
MBP^c^	DnBP	0.84	2.34	0–5.86	0.67	1.87	< LOD to 4.70
MiBP^c^	DiBP	0.12	0.41	0–2.90	0.09	0.33	< LOD to 2.3
MBP + MiBP	DBP	0.99	2.68	0–5.98	0.79	2.15	< LOD to 2.15
MEHHP	DEHP				1.33	9.11	< LOD to 128.5
MEOHP	DEHP				2.00	12.8	< LOD to 158.9
Average^d^	DEHP				1.70	10.72	< LOD to 143.7
MEHP	DEHP	1.32	9.32	0–41.1	2.37	16.8	< LOD to 73.9

LOD, limit of detection.

a *F_UE_* values for MEHP, MEHHP, and MEOHP are 0.059, 0.233, and 0.150, respectively, based on human data from Koch et al. (2005).

b Current U.S. EPA RfDs are 20 (DEHP), 100 (DBP), 200 (BBzP), and 800 (DEP) μg/kg/day (U.S. EPA 2005a, 2005b, 2005c, 2005d).

c Statistically significantly associated with reduced AGI in the Swan et al. (2005) study.

d Average of the exposure estimates using MEHHP and MEOHP.
