# Erratum

**Published:** 2007-02

In Table 2 of the the commentary by Marsee et al. [ Environ Health Perspect 114:805–809 (2006)], the value for the 75th percentile of monobenzyl phthalate (MBzP) was incorrect; the correct value is 0.92.

The authors regret the error.

